# Distal Consequences of Oral Inflammation

**DOI:** 10.3389/fimmu.2019.01403

**Published:** 2019-06-25

**Authors:** Joanne E. Konkel, Conor O'Boyle, Siddharth Krishnan

**Affiliations:** ^1^Faculty of Biology, Medicine and Health, Manchester Academic Health Science Centre, Lydia Becker Institute of Immunology and Inflammation, University of Manchester, Manchester, United Kingdom; ^2^Manchester Collaborative Centre for Inflammation Research (MCCIR), University of Manchester, Manchester, United Kingdom

**Keywords:** periodontitis, adaptive immunity, innate immunity, antigen mimicry, innate cell training, inflammation, commensal bacteria

## Abstract

Periodontitis is an incredibly prevalent chronic inflammatory disease, which results in the destruction of tooth supporting structures. However, in addition to causing tooth and alveolar bone loss, this oral inflammatory disease has been shown to contribute to disease states and inflammatory pathology at sites distant from the oral cavity. Epidemiological and experimental studies have linked periodontitis to the development and/or exacerbation of a plethora of other chronic diseases ranging from rheumatoid arthritis to Alzheimer's disease. Such studies highlight how the inflammatory status of the oral cavity can have a profound impact on systemic health. In this review we discuss the disease states impacted by periodontitis and explore potential mechanisms whereby oral inflammation could promote loss of homeostasis at distant sites.

Barrier sites such as the mouth, gut and skin are interfaces between our inside bodies and the outside world. Maintaining their integrity is crucial to survival. Each barrier has unique requirements and must perform its physiological function whilst maintaining control of commensal microbes and responding to environmental insults and pathogen invasion. The immune system critically promotes barrier integrity in the face of these challenges and is carefully tailored to barrier environments, creating highly-specialized immuno-surveillance networks that police these sites. One area of intense research focus is to understand how tissue-specific signals balance immunity and regulation at barrier sites (1, 2). Elucidating these mechanisms is essential to develop tissue-specific therapies for disease. One barrier at which appropriate immune control frequently fails is the oral barrier. Breakdown of balanced immune responses at the gingiva, a key oral barrier, leads to periodontitis, the most common chronic inflammatory disease of humans, affecting ~50% of the UK population (3). This prevalent disease undermines the tooth-supporting tissues, the periodontium, resulting in impaired dentition and tooth loss.

Specific bacteria have long been thought to drive development of periodontitis (4), however, more recent data indicates that diverse microbial communities are associated with disease (5). Although, triggered in response to the local microbiota, key to the development of periodontitis pathology are defects in the well-balanced inflammatory reactions occurring at the oral barrier. For example alterations in neutrophil recruitment and function as well as increases in T-helper 17 (Th17) cells have been shown to be key in periodontitis development (6–8). Detailed insight and understanding the pathogenesis of periodontitis is vital, as not only does this chronic disease affect a substantial proportion of the global population, but periodontitis has been associated with the initiation, exacerbation, and pathogenesis of a plethora of other inflammatory diseases.

Indeed, recurring inflammation at the oral barrier has been shown to adversely affect systemic health, which may be especially important in individuals with pre-existing conditions. Periodontitis has been routinely associated with a multitude of diseases, ranging from inflammatory to infectious, as well as developmental, cardiovascular, and neurodegenerative conditions. Although aberrant immune responses, heightened inflammation, and oral bacteria are frequently proposed to explain these associations, the exact contribution of periodontitis to the etiology and/or progression of systemic disease is unclear in most instances. What is clear, however, is that pathology in the oral cavity can lead to potentially deleterious consequences for the rest of the body. In this review we will briefly discuss the diseases that have been associated with periodontitis as well as outlining the possible mechanisms by which periodontal inflammation could mediate its deleterious effects at distal body sites. We aim to cover the key mechanisms that have already been suggested but will additionally highlight novel mechanisms by which periodontitis could affect distal inflammatory diseases; taking lessons from inflammation occurring at extra-oral sites.

## From Hearts and Minds to Joints; the Reaches of Periodontitis

To date, perhaps the condition most strongly linked with periodontitis is cardiovascular disease (CVD). An association between periodontitis and CVD was first reported 20 years ago (9). Since, periodontitis has been consistently reported in both epidemiological and experimental studies to have a role in the development and progression of atherosclerotic cardiovascular disease (10–12). Periodontal treatment may also reduce the prevelance of CVD (13), or, at the very least, mitigate some of the risk factors, by improving endothelial function (14), or lowering inflammatory markers (15, 16). Most frequently it is periodontal bacteria that have been proposed to drive CVD, as bacterial species isolated from atherosclerotic plaques are thought to be derived from the oral cavity (17). In particular, viable periodontal bacteria such as *Porphyromonas gingivalis, Streptococcus sanguis, Aggregatibacter actinomycetemcomitans*, and *Tannerella forsythia* have been isolated from atheromas (18–20). These oral microbes are proposed to exert atherogenic effects by accumulating at sites of plaque development and modulating local vascular inflammation (21), with *P. gingivalis* specifically reported to exacerbate atherosclerosis in animal models (22).

As periodontitis is causally linked to CVD, periodontitis is also by association often linked with cerebrovascular disease (23–26), a link that remains uncertain from recent evidence in experimental studies (27). Some epidemiological studies propose that dental infections, including periodontitis, increase the risk of stroke (28–30), although not all indicate an increased risk (31). Periodontal bacteria and their products are associated with systemic inflammation and platelet aggregation, which are known contributors of increased brain damage post-stroke (32).

In addition to CVD, substantial epidemiological and pre-clinical evidence indicates a role for periodontitis in the pathogenesis of rheumatoid arthritis (RA) (33–35). For decades, a link between periodontitis and RA has been proposed, with evidence suggesting that periodontitis can exacerbate or initiate RA, and vice versa. Current models for RA induction include the “two-hit” model; “one” where tolerance is broken and “two” where pathology is established/amplified; periodontitis could contribute to either and/or both of these steps. Recent evidence lends credence to the suggestion that periodontitis could represent the second hit, promoting inflammation and specifically driving RA pathology. Indeed, individuals with chronic RA have a higher incidence of periodontitis, and similarly, the prevalence of RA is higher in periodontitis patients (34). Moreover, RA patients with persistent periodontitis are less responsive to conventional therapeutic interventions (36) and periodontitis treatment can reduce the severity of RA (37). Animal models have also clearly indicated that RA is exacerbated by periodontitis (35, 38). Most research to date has focused on the roles of periodontal bacteria in driving the inflammatory consequences of periodontitis in RA; in fact, DNA from periodontal microbes have been detected in human synovial fluid during RA (33). In particular, *P. gingivalis* has been isolated from joints and is thought to drive RA pathology through molecular mimicry which promotes pathogenic self-reactive T cells that exacerbate disease (discussed further in the next section) (39). Given the pathogenic potential of anaerobic bacteria such as *P. gingivalis*, it is unsurprising therefore that antibacterial treatments (such as ornidazole, ievofloxacin, and clarithromycin) also lessen the severity of RA (40–42), implying oral bacteria could be an important driver of rheumatoid pathology.

More recent evidence has also implicated periodontitis with an increase in cognitive decline in Alzheimer's disease (AD) (43–45). In particular, periodontitis contributed to a six-fold increase in cognitive decline in AD patients which was independent of baseline cognitive state, again possibly driven by modulation of the patients' inflammatory state (46). Pre-clinical findings support this association, highlighting that periodontal bacteria can gain access to the brain in genetically-susceptible mice, contribute directly to pathology by promoting neuroinflammation (47–49), and kill neurons through the activity of extracellular proteases (45).

Given that periodontitis represents an inflammatory disease of the oral mucosa, it is perhaps not surprising that periodontitis is associated with driving pathology at other mucosal surfaces, most notably the gut (50) and the lung (51). Considering that the oral cavity is the access point to both respiratory and gastrointestinal (GI) tracts, it is conceivable that oral inflammation could affect the lung or GI environments. In particular, periodontitis has been proposed to exacerbate inflammatory bowel disease and colorectal cancer. Elevated levels of periodontal bacteria increase the risk of developing pre-cancerous gastric lesions (52), and the oral-derived *Fusobacterium nucleatum* has been shown to directly promote colorectal cancer (53, 54). In addition to potential oncogenic effects, oral bacteria are proposed to contribute to the inflammatory pathology in inflammatory bowel disease (IBD) (50). Patients with IBD are reported to have higher prevalence and extent of periodontitis compared to healthy individuals (55, 56). However, despite some preclinical evidence indicating a possible role for periodontal bacteria in the gut (57, 58), as with the other diseases already discussed, a direct causal association between periodontitis and IBD has not been established. With regard to periodontitis and lung pathologies, while the current evidence is perhaps circumstantial, some clinical and pre-clinical studies have found that periodontal bacteria are capable of directly contributing to lung pathology; *A. actinomycetemcomitans* can cause pulmonary infections of humans either alone or in tandem with *Actinomyces* species (59). Furthermore, *P. gingivalis* has also been isolated with the opportunistic pathogen, *Pseudomonas aeruginosa* in tracheal aspirates from patients with chronic obstructive pulmonary disease (60), and in mice, *P. gingivalis* and *Treponema denticola* have been reported to exacerbate lung pathology (61).

In addition to the aforementioned associations between periodontitis and systemic diseases, there is also some evidence linking periodontitis with obesity (62), diabetes (63), non-alcoholic steatohepatitis (64, 65), and pregnancy complications (66, 67). Periodontitis is more prevalent in diabetics and obese individuals (62, 63) and may contribute to metabolic dysregulation (68, 69). In relation to pregnancy, periodontal bacteria have been implicated in low birth weight, premature birth, and miscarriage (66, 67). Indeed, both *F. nucleatum* and *P. gingivalis* have been shown to colonize the placenta and thought to drive inflammation which is associated with fetal loss in both human and mouse studies (66, 70).

Despite a wealth of information associating periodontitis with adverse impacts on systemic health, evidence causally tying periodontitis to disease development and/or progression is lacking. This is due to a number of issues. In particular presence of oral disease is typically under-reported in individuals with severe health conditions. Unless oral parameters are explicit readouts of an otherwise non-oral disease, then they are likely not considered, and therefore frequently not examined; limiting useful evidence. Nevertheless, multiple animal studies have identified a number of mechanisms by which periodontal inflammation mediates potentially deleterious consequences at body sites away from the oral cavity.

## Mechanisms Identified; How Periodontitis Complicates Disease Processes at Distal Sites

**Figure 1:**
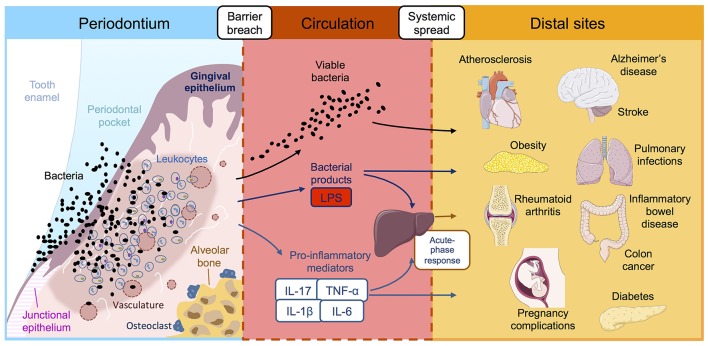
Reported associations between periodontitis and systemic diseases. Periodontitis is initiated by ulceration of the gingival epithelium, bacterial invasion and influx of immune cells, leading to inflammatory damage to the periodontal tissues and destruction of the supporting alveolar bone. This chronic inflammatory reaction leads to leakage of bacterial products, host inflammatory factors and pathogenic oral bacteria into the bloodstream where they are transported to distal tissue sites. Once in the systemic circulation, periodontially-derived products have the potential to adversely affect a multitude of systemic diseases, either directly *in situ* or indirectly via amplification of the systemic inflammatory response. LPS, lipopolysaccharide; IL, interleukin; TNF-α tumor necrosis factor-alpha.

Most of the evidence linking periodontitis and systemic disease centers around the physical dissemination of periodontal bacteria and/or immunogenic factors via the circulation (71, 72). Chronic inflammation coupled with a richly vascularized periodontium leads to ulceration of the oral epithelial barrier, and consequently, greater access for pathogenic microbes and their products to the bloodstream. Generally, the chronic systemic distribution of oral bacteria-derived products converges at the point of an altered state of immunity, achieved either through subversion of host defenses, or prolonged and/or enhanced inflammatory responses. These changes are thought to amplify the overall threat that periodontitis poses to the host (Figure 1).

### The Virulence of Periodontal Bacteria; Contributions to Systemic Disease

The ability of oral bacteria to invade and thrive in non-oral sites could unbalance homeostatic tissue responses in their new home and potentially contribute to disease. Since oral pathogens thrive within anaerobic periodontal pockets (73), the virulence factors that enable successful oral colonization may also render them capable of thriving at other sites. Indeed, prominent oral pathogenic bacteria including *F. nucleatum, P. gingivalis*, and *A. actinomycetemcomitans* have been detected in a multitude of extra-oral tissue sites, including the lung, heart, gut, placenta, and inflamed joints (33, 47, 49, 51, 66, 74, 75). Furthermore, all aforementioned species are capable of invading epithelial and endothelial cells (76), and specifically, *A. actinomycetemcomitans* can spread to neighboring cells, disseminating via membrane-bound vacuoles endocytosed by adjacent host cells (74). Indeed, even a site like the brain, with a restrictive blood-brain barrier, is not protected from invasion by oral bacteria; oral *Treponema* spirochetes have been found in human AD brains and in branches of the trigeminal nerves, indicating a potential route of access to the demented brain (77). In addition to a remarkable invasiveness potential, periodontal bacteria possess an extensive suite of virulence factors which facilitate colonization at extra-oral sites; fimbriae, adhesion molecules, and capsules are just some components of an arsenal that equip oral bacteria with the capability to persist outside the periodontium (78).

Periodontal bacteria also utilize sophisticated immune subversion mechanisms which can undermine the host response and thereby enable persistence at extra-oral sites, and include evasion of complement-mediated killing (79, 80), disarming leukocyte responses (81, 82), inhibition of lymphocyte activity (83), and modulation of toll-like receptor (TLR) signaling (81). The details of the immune evasion strategies employed by oral bacteria are beyond the scope of the current review, but have been well-outlined previously (78). While most evidence to date has been carried out in the context of the oral cavity, some studies have endeavored to address whether these subversive strategies can be employed at extra-oral sites, mostly in the context of atherosclerosis (84–86). In a similar regard, recent evidence from both pre-clinical models and human patients, suggest that oral bacteria can “hijack” immune cells (87, 88), enabling systemic dissemination from oral foci while simultaneously evading killing.

Independently of employing direct immune evasion strategies, periodontal bacteria also disrupt homeostatic processes, which has the potential to adversely impact systemic health. In this regard, *P. gingivalis* and *S. sanguis* can induce platelet aggregation (89, 90), which may contribute to an increased risk of infarction. *P. gingivalis* is also capable of modifying low-density lipoprotein which promotes foam cell formation (91, 92), a pathogenic feature of atherosclerotic disease. Extracellular proteases from *P. gingivalis* can kill neurons *in vitro*, thereby aggravating cognitive dysfunction in mouse models of AD (45), and in the context of metabolic disease, *P. gingivalis* can promote glucose intolerance and insulin resistance in mice fed a diabetogenic diet (57). In the gut environment, *F. nucleatum* can bind to oncogenic cells via its Fap2 protein, promoting colorectal cancer (53). In addition, oral *Klebsiella* species have been shown to promote T-helper 1 inflammatory responses in the gut, contributing to GI inflammation in mice (50). In all these cases tissue homeostasis is disrupted potentially laying the foundation for disease initiation and/or further amplification.

Well-documented within the oral cavity (78), inter-species microbial synergy could support oral bacteria survival at sites outside the oral cavity. Although further research is needed, studies have postulated a role for extra-oral microbial synergy by periodontal bacteria; for example *P. gingivalis* promotes the ability of *P. aeruginosa* to invade and persist in respiratory epithelial cells *in vitro* (93, 94), and the temporal dynamics of *F. nucleatum* and *P. gingivalis* colonization which mutually benefits survival in the oral cavity are also posited to do the same in the gut and contribute to colorectal tumorigenesis (95).

It is important to note, that while the aforementioned strategies relate to viable bacteria promoting their own persistence at extra-oral sites, chronic presence of oral bacteria in the systemic compartment will provoke an immune response, regardless of viability. This undoubtedly escalates the burden that host defenses are tasked with as large numbers of oral commensals are transported into the bloodstream during periodontitis. This may explain why chronic bacteremia as a result of periodontitis poses an important threat, especially when periodontal pathogens themselves are usually found in low numbers (73).

### Leakage of Microbial and Host Factors

While destruction of the oral basement membrane facilitates increased leakage of microbes from the periodontium, so too are locally-produced host and microbial factors flushed into the systemic circulation. Furthermore, dental procedures (scaling, extractions, root planning etc.) as well as habitual brushing, flossing, and mastication all increase the rate of blood contamination (96) and in turn, aggravate the systemic inflammatory response. In this regard, lipopolysaccharide (LPS) from Gram negative bacteria such as *P. gingivalis* has been given the most attention. Although more weakly immunogenic than it's *E. coli* counterpart, blood-borne LPS from *P. gingivalis* can induce inflammation *in vivo* (97–99) and can also directly contribute to the pathogenesis of atherosclerosis in pre-clinical models (100–102). In patients with severe periodontitis, even gentle mastication can lead to elevated LPS in the circulation (71), and, when taken together, suggests that systemic leakage of oral-derived LPS is an important determinant of further complications outside the periodontium.

In addition to microbial factors, chronic oral inflammation can also result in sustained and increased levels of host inflammatory mediators in the circulation, which is a trigger for the acute-phase response. Indeed, the acute-phase reactants, interleukin (IL)-6, C-reactive protein (CRP), haptoglobin, fibrinogen, serum amyloid A, and serum amyloid P are elevated in periodontitis patients (103–107). Prolonged or excessive activation of the acute-phase response is associated with systemic pathology such as sepsis and ischaemia-reperfusion injury (108–110), as well as cardiovascular disease (111). As such, one way in which periodontitis could contribute to systemic disease may be via modulation of the acute phase response.

Moreover, there are reports of serum increases in levels of the pro-inflammatory cytokines IL-6 (105), IFN-γ (112), as well as TNF-α and IL-17 in patients with generalized aggressive periodontitis (113, 114); findings from animal models of periodontitis also support a heightened systemic inflammatory profile (58, 115, 116). Sustained elevation of inflammatory cytokines in the serum is a well-documented driver of cerebrovascular and cardiovascular disease in humans and in animal models (105, 117, 118). Although evidence directly tying periodontitis to systemic disease via increased circulating cytokines is lacking, some studies, both clinical and pre-clinical, have reported that periodontitis-induced increases in serum IL-6 and CRP levels can lead to endothelial dysfunction (115, 119). Thus, transit of immunomodulatory factors from an inherently leaky oral focus of inflammation into the circulation could potentially alter the course of systemic disease progression and represents a potential avenue by which periodontitis could contribute to pathology at distal sites.

### Antigen Mimicry

In general, periodontal bacteria can actively modulate the innate immune response and their products can provoke the inflammatory response, whether this is at oral or extra-oral sites. Aberrant immunity represents a general explanation for the adverse effects of periodontitis on systemic diseases, but perhaps the most robust evidence for a direct role of periodontal bacteria in a specific disease setting is the ability of *P. gingivalis* to promote RA pathology via molecular mimicry (39, 120, 121). Citrullination of peptides, and consequent autoantibody generation, is a pathogenic hallmark of RA, and importantly occurs prior to disease onset as well as correlating strongly with disease severity (122). *P. gingivalis* is unique in that it can citrullinate proteins via its own enzyme, peptidylarginine-deiminase (PAD), and therefore is a direct contributor to the production of pathogenic antibodies, driving auto-reactive T cells to promote inflammatory synovial destruction (39, 120, 121). In this regard, PAD-deficient *P. gingivalis* strains fail to exacerbate RA severity in mice (39). More recently, *A. actinomycetemcomitans* has also been shown to promote citrullination of peptides. Here, rather than possessing enzymatic capacity themselves, *A. actinomycetemcomitans* has been shown to trigger dysregulated activation of citrullinating enzymes in neutrophils (123).

*P. gingivalis* has also been reported to elicit antigen mimicry in the context of CVD and pregnancy complications. Cross-reactive bacterial epitopes mimic host cardiolipin and induce specific autoantibodies that have been associated with atherosclerotic thrombosis and adverse pregnancy outcomes (70, 124). Moreover, elevated levels of cardiolipin-specific antibodies are found in the gingival crevicular fluid and serum of periodontitis patients (124, 125). Antigen mimicry is further suggested to link oral and atherosclerotic disease through the cross-reactivity of heat-shock proteins (HSPs) with oral bacterial components (126). HSPs are associated with enhanced atherosclerotic development (127) and *P. gingivalis* GroEL proteins are highly homologous to HSP60 (126, 128) and thus capable of contributing to disease pathology.

### Aberrant Immune Responses

In addition to chronic systemic elevation of host inflammatory mediators, specific changes in host immunity have been reported to link periodontitis and distal diseases. This point is nicely illustrated in studies where genetic alterations in host immunity confer an increased risk to RA in the presence of a periodontitis-dependent stimulus. Specifically, in human-leukocyte antigen (HLA)-DRβ1 humanized mice, which are susceptible to RA, exposure to oral *P. gingivalis* results in the generation of anti-citrullinated protein antibodies; something which does not occur in wild-type mice (129). This suggests that changes in the host immune response can underpin the systemic impact of periodontitis.

In a similar sense, changes in inflammatory genes can not only confer susceptibility to periodontitis, but can also account for the increased risk of adverse systemic effects due to periodontitis. IL-17 is a major driver of disease in periodontitis (130), RA (131), as well as type I diabetes mellitus (132). Indeed, genetic polymorphisms in IL-17A have been suggested to enhance the impact of periodontitis on type I diabetes (133), indicating that a failure of adequate host immunity during periodontitis can lead to worsening of other non-oral diseases.

IL-17 is also implicated in mediating the crosstalk between periodontitis and RA. *P. gingivalis* induced periodontitis enhances the severity of articular injury during experimental arthritis; this aggravation of arthritis did not occur in IL-17RA-deficient mice (134). Additionally, the levels of IL-17 produced in response to *P. gingivalis* and *Prevotella nigrescens* correlates with the intensity of arthritic bone destruction (135). Furthermore, IL-17 can promote even more distant effects outside gingiva and joint, as mice with experimental RA orally-infected with *P. gingivalis* have raised serum IL-17 levels, increased Th17 cells in mesenteric lymph nodes, and exhibit shifts in the composition of the gut microbiome (136). Together, these studies indicate that IL-17 may be a mediator of the deleterious effects of periodontitis at distant sites, aggravating pathology in a number of disease contexts. Nevertheless, it is important to remember that during periodontitis, elevated production of IL-17 is due to Th17 cells, cells which produce IL-17 in direct response to the dysbiotic microbiota (130). Thus, even if aberrant host immunity is ultimately the driver of local and systemic pathology, this only occurs because of microbial signals. For example, in the absence of TLR2 in antigen-presenting cells, Th17-dependent IL-17 production is impeded, highlighting that initial microbial sensing is an important initiator of dysregulated host immunity in periodontitis (135).

Altered innate immune responses during periodontitis have also been suggested to mediate some dangerous crosstalk between oral and extra-oral sites. Neutrophils play key roles in local periodontitis pathology (137), and also in multiple disease states in which periodontitis is associated (134, 138). Neutrophil responses during periodontitis could promote systemic exacerbations, especially in the context of RA, since neutrophils release PADs and neutrophil extracellular traps (which contain chromatin and granular contents to bind and kill pathogens) which citrullinate proteins in the joints (138). Furthermore, the neutrophil-recruiting complement component C5a is produced during periodontitis and has been shown to exacerbate RA progression (139). In addition to altered neutrophil responses, there is also evidence to suggest that aberrant monocyte responses can promote systemic dysfunction. Specifically, ligature-induced periodontitis in rats alters circulating monocytes, rendering them more adherent to aortic vascular endothelial cells via VCAM-1 binding, and thereby increasing the risk of atherosclerosis (140). Periodontitis-activated monocytes could also prime Th17 cells for enhanced IL-17 production (141), suggesting that a dysregulated interplay between innate and adaptive immune networks may contribute to the risk of complications at distant sites.

Ultimately, systemic complications due to periodontitis are the product of crosstalk from the host as well as microbes. Untangling the exact contribution of each component has so far been extremely difficult and complicated by recent evidence suggesting that periodontitis can drive changes in the composition of the gut microbiome (58, 142). Gut dysbiosis in itself is well-known to cause profound changes in host immunity, and consequently is a contributor to a range of diseases (143). Thus, not only can periodontitis directly lead to dysregulated host immunity, but it can alter microbial communities locally as well as distally and indirectly impact immune responses. This emphasizes the complex nature of how periodontitis can affect systemic diseases. It is important to reiterate that even though we have described specific instances whereby altered host immunity or microbial activities clearly predispose to detrimental systemic effects, they are only one part of the overall picture. Adverse distal effects of periodontitis are most likely the result of a dynamic interplay between microbial signals and aberrant host responses.

## Inter-Organ Immunity; Long Range Control of Immune Responses

As outlined above, in the setting of periodontitis, oral microbes themselves, their products, or indeed, host immune mediators are all capable of eliciting changes to systemic immunity, by amplifying inflammation, subverting innate immune defenses, and promoting autoimmunity; all of which have the net effect of promoting systemic complications and disease development. Despite this, the idea that inflammation at one site can lead to alterations in immune responsiveness at another is not new, and indeed has been demonstrated in a number of settings away from the oral cavity. In particular, in 1978 the idea of the common mucosal immunologic system was proposed (144), suggesting mucosal sites communicate to protect the body from pathogenic challenge. Subsequently, increasing evidence has emerged suggesting that immune activation at one mucosal compartment directly impacts immunity at a distal mucosal site (145–148). The oral mucosal barriers of the mouth would certainly form a constituent of these collective mucosae, and thus immunological cross-talk between the oral barrier and distant mucosal sites has perhaps long been proposed in healthy if not in pathological settings. In addition to this mucosa-to-mucosa cross-talk, inflammation at non-oral barrier sites has also been associated with rheumatoid arthritis and joint pain, skin inflammation, as well as pathologies of the central nervous system (CNS). Thus, that inflammation at one site can effect inflammatory processes at another is not unexpected, but periodontitis is unique in the plethora of pathologies it has been reported to impact; perhaps a result of the inherent leakiness of the oral barrier.

**Figure 2:**
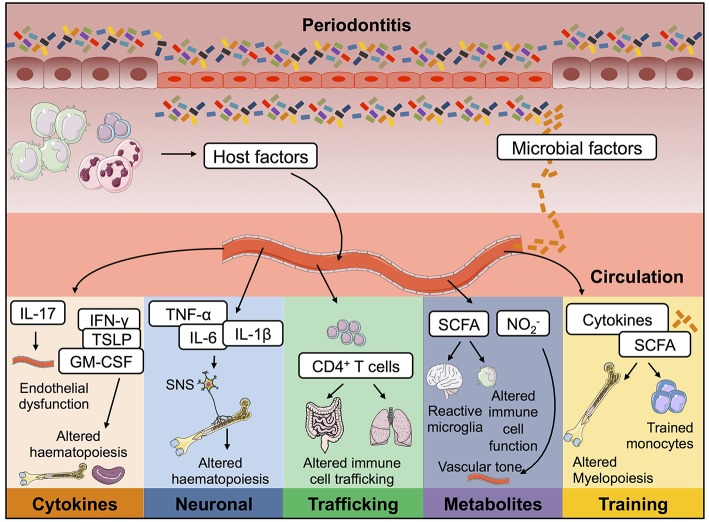
Possible mechanisms by which periodontitis contributes to inflammation at distal tissue locations. During oral inflammation both host- and microbial-derived factors could contribute to inflammatory pathology at extra-oral sites. Passage into the blood stream would give these immune-modulators access to a plethora of body compartments and therefore mediate effects on many body systems. Periodontitis could therefore contribute to the pathophysiology of other diseases in the following ways; (i) lead to endothelial dysfunction; (ii) drive changes to haematopoiesis; (iii) promote activation of T cells capable of trafficking to other mucosal barriers; (iv) result in changes to circulating immuno-modulatory microbial-derived metabolites; and (v) cause innate immune cell training. IL, interleukin; TNF-α, tumor necrosis factor α; PGE_2_, prostaglandin E_2_; SCFA, short chain fatty acids; ${\text{NO}}_{2}^{-}$ nitrite.

Thus, far we have discussed possible mechanisms of cross-talk which have already been shown to contribute to how oral inflammation promotes a plethora of systemic diseases. Taking our cues from data that does not examine the oral barrier; we will now discuss immunological mechanisms that have been described whereby inflammation at one site affects immune control at another. We will outline possible mechanisms that periodontitis could hijack to exacerbate inflammation at distal sites such as the CNS and bone marrow, discussing studies that do not focus on periodontitis but other settings of acute, chronic or mucosal barrier inflammation. Exploring established and emerging concepts we will consider the relevance of these long-range signals to the distal inflammatory effects mediated by periodontitis (Figure 2).

## Possible Mechanisms by Which Periodontitis Could Contribute to Distal Diseases

### Cytokine Communication

As already discussed, the levels of certain cytokines have been shown to increase in the serum of periodontitis patients. Some of these elevated inflammatory cytokines could be mediating distal effects in the bone marrow. A number of studies have implicated cytokines such as IL-12, granulocyte-macrophage colony-stimulating factor (GM-CSF), thymic stromal lymphopoietin (TSLP), and IL-17 as candidates that can mediate long-range signals that influence the fundamental process of haematopoiesis (149–155). For example, produced in the gastrointestinal tract during inflammation, IL-12 is also detected within the blood and can be sensed in the bone marrow, modulating immune cell output. Monocytes primed for regulatory functions are generated upon instruction by natural killer (NK) cells in the bone marrow which sense inflammation-driven systemic IL-12 and release interferon (IFN)-γ (150). In line with this cytokines such as GM-CSF and TSLP have also been shown to mediate effects distal from their site of generation during settings of auto-inflammation. In another example, Siracusa et al. (155) reported that systemic TSLP can elicit extramedullary haematopoiesis in the spleen. This resulted in the expansion of progenitors with an intrinsic preference to differentiate into granulocytes; these immune cells promoted the development of allergic airway inflammation. Such studies demonstrate that localized inflammation results in the systemic elevation of cytokines that feed-back to the bone-marrow, subsequently altering the quality and/or quantity of the immune cells generated.

In conjunction with relaying signals to the bone marrow, systemic cytokines could directly modulate other distal tissues such as the CNS. It has been recently demonstrated that a gut-initiated Th17 response is capable of inducing cognitive decline through the effects of systemic IL-17 on the cerebrovasculature, inducing endothelial dysfunction (152). As cytokine levels are altered systemically in periodontitis, impacts of these on bone marrow output, as well as the CNS, could be easily envisaged and future work should examine changes to these compartments in patients and in pre-clinical models of periodontitis. With specific relation to changes to the bone marrow compartment, such fundamental alterations in immune cells could explain why periodontitis can impact the pathogenesis of such a variety of other diseases.

### Neuronal Signals

As well as cytokines, neuronal signals, particularly those of the autonomic nervous system have also emerged as critical regulators that can relay long-range signals to the haematopoietic compartment during homeostasis and inflammation (156–160). As a result, it becomes essential to consider the role of these neuronal pathways during periodontal inflammation as their activation could well-contribute to the systemic inflammatory effects of this disease.

The concept of neuro-immune cross-talk has emerged from elegant demonstrations of the dynamic interactions between the nervous and immune systems in both homeostasis and non-periodontal disease that has reformed our understanding of these seemingly disparate systems (161–170). A number of studies have demonstrated that the bone marrow haematopoietic compartment receives rich sympathetic innervation (156, 171, 172) and that adrenergic signaling is critical in modulating haematopoietic output (156, 173), its circadian oscillations (171), aging (157) and ability to regenerate in contexts of injury (158). For example, the interaction of noradrenaline with the β_2_ adrenergic receptor have been shown to be indispensable for Granulocyte colony-stimulating factor (G-CSF)-mediated mobilization of haematopoietic stem and progenitor cells (HSPC), through the downregulation of the bone marrow anchor, CXCL12 (156). The ability of the sympathetic nervous system to exert diverse effects on the bone marrow haematopoietic niche stems from the transduction of signals via distinct β-adrenergic receptor sub-types (157, 171, 173). Intriguingly, the capacity of the autonomic nervous system to tune the bone marrow HSPC compartment is not restricted to neuronal signals. Non-myelinating schwann cells in the bone marrow are thought to play an integral role in maintaining HSPCs and their quiescence through their ability to contact HSPCs and activate latent transforming growth factor (TGF-β) (174). These networks are also thought to be important in disease as inflammation could disrupt the neuro-immune crosstalk. Indeed, there is a large body of evidence that has implicated sympathetic over-activation following ischaemic stroke in driving alterations in systemic immunity (175–177), in part, mediated through alterations in haematopoiesis in the bone marrow (159).

The ability of the nervous system to shape immune function is not limited to effects on haematopoiesis as neuropeptides derived from the enteric nervous system have also emerged as key players that shape immune function in health and disease (170, 178–183). Critical in driving group 2 innate lymphoid cell (ILC2)-mediated type 2 inflammation, the neuropeptide, neuromedin U has been shown to augment type-2 responses and therefore amplify allergic inflammation (182, 183). Recently, cytokines have emerged as candidates that are capable of directing immune function through effects on the nervous system (184). In this context it can be postulated that inflammation could modulate immune functions through alterations in neuronal signaling mediated by cytokines. As a result, it can be envisaged that the systemic leakage of cytokines during periodontal disease could engage autonomic and enteric neuronal circuits in the gut or bone marrow that could exacerbate systemic inflammation.

### Trained Innate Immunity

Thus, far, we have considered how cytokine and neuronal signals altered in periodontal disease may mediate immune changes that could contribute to altered inflammatory responses at non-oral sites. Here, we shall delve into a more fundamental concept, how the sequence of signals experienced by innate cells are critical in directing their effector function, a concept historically confined to the realms of adaptive immunity. Although this has yet to be explored in settings of periodontitis, we shall discuss how through its myriad of effects, periodontal disease could subvert evolutionarily conserved pathways that serve to enhance and diversify the repertoire of innate responses to pathogenic challenges.

Traditionally it is considered that innate immune cells, such as monocytes, acquire effector functions upon recruitment to inflammatory sites, experiencing local signals that drive functional adaptation and local differentiation. However, it has now emerged that the terminal effector function of mononuclear phagocytes can be imprinted by infection, injury-driven cytokines or microbe-derived metabolites in distal tissues such as the bone marrow, altering the monocyte compartment as a whole (150, 185, 186). These studies add to the growing body of evidence (187) that illustrates the concept of trained innate immunity, a primitive means to tune the monocyte network to protect against pathogenic challenges.

Recent studies have illustrated that innate immunity can be trained to protect against subsequent challenges through the emergence of short-lived distinct myeloid populations that have a modulated, usually enhanced capacity to respond to pathogenic challenges. For example, monocytes, whilst one of the first responders to infection or injury possess an intrinsic inability to initiate pathogenic-specific responses like T cells or take on sub-specializations like tissue-specific macrophages (188, 189). In order to facilitate an expeditious and effective immune response during infection, it is thought that the host replaces the monocyte compartment as a whole through targeted alterations in haematopoiesis involving epigenetic rewiring (150, 186, 190, 191). Critically, it is distinct from emergency haematopoiesis which occurs in response to infection (192) as it tunes immunity by altering the functional responsiveness of the innate cells, specifically monocytes, generated.

Although trained immunity can have a positive effect in settings of infection, alterations in innate cell function could well mediate disastrous consequences in settings of auto-inflammation and specifically, contribute to the distal effects of periodontitis. Diverse stimuli have been shown to be capable of imparting monocyte training, including metabolites such as short chain fatty acids (SCFAs) (185) and mevalonate (193), and infection-driven cytokines such as IFN-γ (150). Whilst it is thought that this immune “memory” is maintained through distinct alterations in haematopoiesis (190), intriguingly, innate immune cells are capable of being trained at multiple stages, from the point of genesis (150, 185, 190) to the point of maturity (194). For example, studies (150, 185, 190) have shown that microbial products such as β-glucan or SCFAs and cytokines such as IFN-γ can modulate myelopoiesis resulting in the production of altered myeloid precursors as a means to imprint immunity against pathogens or in response to inflammation at mucosal sites such as the lung or gut. Whilst mature monocytes can also be trained by similar stimuli such as β-glucan, the training is thought to be epigenetically imprinted and consequently confers an enhanced capacity to respond to subsequent pathogenic challenges (194, 195). Unlike alterations in haematopoiesis, epigenetic signatures serve to impart immune “memory” to mature cells that have exited the haematopoietic developmental programme. It can thus be inferred that these mechanisms act in synergy, modulating developing as well as mature monocytes, replacing the monocyte compartment of the host.

It is conceivable that the systemic dissemination of cytokines, microbial products, and metabolites that occurs during periodontitis, could drive innate immune cell training. Unlike the changes to global haematopoiesis discussed in the previous sections, periodontitis could drive fundamental changes to monocyte function, resulting in the specification of immunogenic phenotypes in monocytes generated in the bone marrow. This could have catastrophic consequences as the generation of trained innate immune cells with altered immunogenic potential could well be a key driver of aberrant inflammation at extra-oral sites. Indeed alterations in peripheral blood monocytes have been noted in periodontitis patients (196–199), potentially suggesting innate immune cell training occurs in this disease. Whether trained epigenetic changes underpin these altered functions remains to be determined. Such fundamental changes to immune cell function, if they do occur in periodontitis, would likely impact subsequent and on-going immune processes, irrespective of their anatomical location.

### Trafficking of Activated T Cells

Usually a consequence of alterations in the cytokine milieu, altering immune cell trafficking is also an effective means to modulate immunity at a distal site. This concept also contributes to the idea of a common mucosal immune system, extending immune responses beyond compartmentalized tissues (200, 201). For example, it has emerged that infection-driven inflammation in the lung is capable of instructing CCR9^+^CD4^+^ T cell recruitment from the lungs to the gut (145). This recruitment of activated T cells to the gut resulted in intestinal injury and dysbiosis, driven by production of IFN-γ and IL-17A. Thus, T cells primed at one mucosal site could drive pathology when found at another. Moreover, intra-nasal delivery of antigens is capable of inducing protective immunity against an enteric *Salmonella* challenge by activating lung dendritic cells (DCs) that promote the homing of CD4^+^ T cells to the gastrointestinal tract through the up-regulation of the integrin α4β7 and CCR9 (146), again highlighting lymphocyte trafficking between mucosal barriers.

In fact, when considering the cross-talk between the oral barrier and distal tissues, it is particularly salient to probe mucosal sites, after all it is well-established that oral vaccination induces protective immunity at distal mucosal barriers (147, 202, 203), indicating responses primed orally are effectively recalled at other barriers. Indeed many antigens encountered in the oral cavity, including those of both food- and bacterial-origin, are subsequently seen in the gastrointestinal tract or lung. As exposure to these antigens will change during oral inflammation, it raises the possibility that altered T cell priming and trafficking could link periodontitis with diseases of the gut and lung.

### Commensal Bacterial Metabolites

It is now well-established that commensal-derived signals play crucial roles in shaping both local immune networks at barrier surfaces, as well as distal tissue sites like the brain (1). This is a relationship that is fraught with peril as a nuanced system of checks and balances are required to limit invasive flora whilst restraining overtly inflammatory responses and allowing the host to reap the benefits of housing a commensal community. As a consequence of this intimate host-microbe relationship, it is unsurprising that shifts in the microbial communities inhabiting barrier surfaces are associated with disease states during inflammation, injury and aging (204–207). In this context, metabolites generated by the gastrointestinal microbial community have been demonstrated to calibrate homeostatic and anti-microbial immune networks *in situ* (208–211). Importantly, not only are these metabolites important for shaping local gastrointestinal intestinal immunity but studies have illustrated that microbe-derived metabolites are capable of being deployed as long-range signals, modulating inflammation at distal sites such as the CNS (212–214) and lung (185, 215, 216). These commensal-derived keystone metabolites thus calibrate immune responsiveness and alteration in the systemic levels of these products has been shown to contribute to immune dysfunction. A number of studies have shown that microbial metabolites from the gut such as the SCFA butyrate play an important role in limiting pathology and promoting the resolution of pulmonary infections through diverse effects on innate and adaptive immunity (185, 217–219). Although not studied to anywhere near the same extent, shifts in the oral commensal community could also contribute to systemic inflammation through the alteration of key metabolites. Focusing on SCFA, the oral commensal community has been shown to shift toward a more butyrate producing community during inflammation (220–223). Whether this leads to subsequent changes in systemic SCFA has yet to be explored, but if so would likely impact peripheral immunity. In addition a key metabolite generated by oral commensals is nitrite, as the oral community reduces nitrates (224–226), generating this metabolite which has vasoactive effects (227–229). Thus, changes in the oral bacterial community during periodontitis could impact systemic levels of nitrite which would have profound effects on systemic vascular tone (229). These concepts await exploration in the context of periodontal disease but again could contribute to the systemic pathologies that are associated with periodontitis.

## Concluding Remarks

Data from pre-clinical animal models and epidemiological studies indicate strong associations between the presence of periodontitis and amplification of plethora of diseased states ranging from joint inflammation to cognitive decline. Despite this, only a few detailed mechanisms have emerged outlining how periodontitis mediates these deleterious effects. As discussed in this review, it is not difficult to envisage possible periodontitis-induced mediators that could be driving distal inflammatory consequences; what remains now is to better delineate these mechanisms and definitively establish cause-and-effect relationships during periodontitis. This can be achieved through well-designed animal experiments utilizing cutting-edge technologies to establish systemic and tissue-specific changes during periodontitis. Moreover, large scale, well-powered, human studies should be undertaken to assess the impact of periodontitis treatment on extra-oral disease pathology. This should be done alongside the careful clinical evaluation of oral parameters in patients with diseases associated with periodontitis, to generate detailed insight into the oral health of these patients. Better understanding of the cross-talk between the oral barrier and distal sites could support a step-change in the clinical treatment of many diseases. Not only could oral health parameters be employed to stratify patients and/or monitor disease progression but in some cases it may emerge that aggressive intervention to improve oral health could mediate dramatic improvements in a plethora of life-limiting diseases.

## Author Contributions

CO reviewed data and wrote the sections regarding the systemic consequences of periodontitis and already postulated mechanisms driving these consequences. SK reviewed data and wrote the sections regarding possible mechanisms whereby periodontitis drives its systemic inflammatory risks. JK supervised the review process, wrote, and compiled the manuscript.

### Conflict of Interest Statement

The authors declare that the research was conducted in the absence of any commercial or financial relationships that could be construed as a potential conflict of interest.
